# Restriction of HIV-1-based lentiviral vectors in adult primary marrow-derived and peripheral mobilized human CD34+ hematopoietic stem and progenitor cells occurs prior to viral DNA integration

**DOI:** 10.1186/s12977-016-0246-0

**Published:** 2016-03-05

**Authors:** Daniel O. Griffin, Stephen P. Goff

**Affiliations:** Department of Biochemistry and Molecular Biophysics, Columbia University Medical Center, HHSC 1310c, 701 West 168th Street, New York, NY 10032 USA; Division of Infectious Diseases, Department of Medicine, Columbia University Medical Center, New York, NY 10032 USA; Howard Hughes Medical Institute, Columbia University Medical Center, New York, NY 10032 USA; Department of Microbiology and Immunology, Columbia University Medical Center, New York, NY 10032 USA

**Keywords:** HIV, Restriction, Lentivirus, Integration, 2-LTR, CD34+, Stem cells, Progenitor cells

## Abstract

**Background:**

Gene therapy is currently being attempted using a number of delivery vehicles including lentiviral-based vectors. The delivery and insertion of a gene using lentiviral-based vectors involves multiple discrete steps, including reverse transcription of viral RNA into DNA, nuclear entry, integration of viral DNA into the host genome and expression of integrated genes. Transduction of murine stem cells by the murine leukemia viruses is inefficient because the expression of the integrated DNA is profoundly blocked. Transduction of human stem cells by lentivirus vectors is also inefficient, but the cause and specific part of the retroviral lifecycle where this block occurs is unknown.

**Results:**

Here we demonstrate that the dominant point of restriction of an HIV-1-based lentiviral vector in adult human hematopoietic stem and progenitor cells (HSPCs) from bone marrow and also those obtained following peripheral mobilization is prior to viral DNA integration. We specifically show that restriction of HSPCs to an HIV-1-based lentiviral vector is prior to formation of nuclear DNA forms.

**Conclusions:**

Murine restriction of MLV and human cellular restriction of HIV-1 are fundamentally different. While murine restriction of MLV occurs post integration, human restriction of HIV-1 occurs before integration.

## Background

Gene therapy, the ability to replace an abnormal gene with a functionally correct copy or to introduce a novel gene for therapeutic purposes, holds great potential for the improvement of human health [1]. Safe and effective gene therapy is being pursued through careful selection of viruses as delivery vehicles and genetic modifications of the original virus [2]. Gene therapy is currently in progress with success in a number of patients with a range of genetic disorders such as immunodeficiency due to adenosine deaminase deficiency [3], Wiskott–Aldrich syndrome [4], metachromic leukodystrophy [5], X-linked severe combined immunodeficiency [6], β-thalassemia [7], sickle cell anemia [8, 9] and X-linked adrenoleukodystrophy [10]. Gene therapy trials are currently ongoing for many other intractable genetic diseases as well as malignancies, arthritis, congestive heart failure, Parkinson’s disease, Alzheimer’s disease and macular degeneration [8].

Despite the ability of lentiviruses to successfully infect non-dividing cells, HIV-1 infection of human hematopoietic stem cell and progenitor cells (HSPCs) is inefficient as compared to activated T cells unless HSPCs are prestimulated [2, 11–13]. An augmentation of successful lentiviral infection of human HSPCs is seen with high multiplicity of infection (MOI) exposures and treatment of human stem cells with multiple cytokines such as interleukin-3 (IL-3), interleukin-6 (IL-6), interleukin-7 (IL-7), stem cell factor (SCF), Flt3 ligand (FLT3L), and thrombopoietin (TPO), with proteasome inhibitors, with targeted siRNA blockade of the cell cycle quiescent factor p21, or with cyclophilin A, rapamycin and cyclosporine [4, 5, 8, 14–21]. Unfortunately, exposure of HSPCs to combinations of cytokines prior to exposure to lentivirus at a high (MOI) decreases the multipotency of HSPCs as evidenced by diminished engraftment potential in murine models [18].

Some investigations suggest the innate response and a type I interferon response may be instrumental in restriction of lentiviruses in certain cell types [22–24]. Other investigations have suggested a limited role of interferon-stimulated genes in the restriction of HIV-1 and related vectors in the human system [25, 26]. It is also unclear whether the restriction of lentiviruses in HSPCs is due to a specific restriction factor such as TRIM5α, as has been found in other cell types, or to the absence of a specific essential co-factor of infection [27–29]. When human HSPCs are pre-stimulated with cytokines a large number of genome-wide modifications occur that allow for successful lentiviral transduction, but the essential changes responsible for this transformation remain unexplored [30].

Although several interventions increase the efficiency of lentiviral transduction, the point in the retroviral replication cycle at which retroviruses are blocked in adult human HSPCs remains unknown. Whether this involves a pre-integration block or is due to transcriptional silencing of integrated proviruses has not been studied in detail and is critical in determining whether murine models and results can be extrapolated to human cells [27, 31–34]. An understanding of the molecular mechanisms used in restriction by HPSCs may allow us to successfully transduce human HPSCs without decreasing their pluripotency, diminishing their repopulating potential or their ability to engraft [35]. We sought to identify the specific point in the retroviral cycle where restriction occurs as a first and important step in understanding the molecular basis of this restriction. Our study evaluates infection of primary adult marrow-derived and peripheral mobilized human CD34+ cells, a mixed population composed of true human HSCs (Lin^−^34^+^38^−^90^+^45RA^dim^) as well as several hematopoietic progenitor cell (HPC) types, with a VSV-G pseudotyped full-length nef-negative HIV-1-based vector.

## Results

### Transduction of primary adult human marrow-derived CD34+ cells by lentiviral vectors is inefficient at low MOI

We investigated the efficiency of lentiviral transduction of primary adult human marrow-derived CD34+ cells under various conditions. We performed each experimental replicate independently with primary adult human marrow-derived CD34+ cells from multiple different donors. CD34+ cells were cultured in serum free VIVO-X 20 media. The cells were pre-incubated for 24 h with no cytokines, FLT3L, SCF, TPO, or all three cytokines (“FST” cocktail). Cells were then exposed to vector virus by spinoculation, left in culture with virus for 72 h at 37°, and evaluated using flow cytometry.

**Fig. 1:**
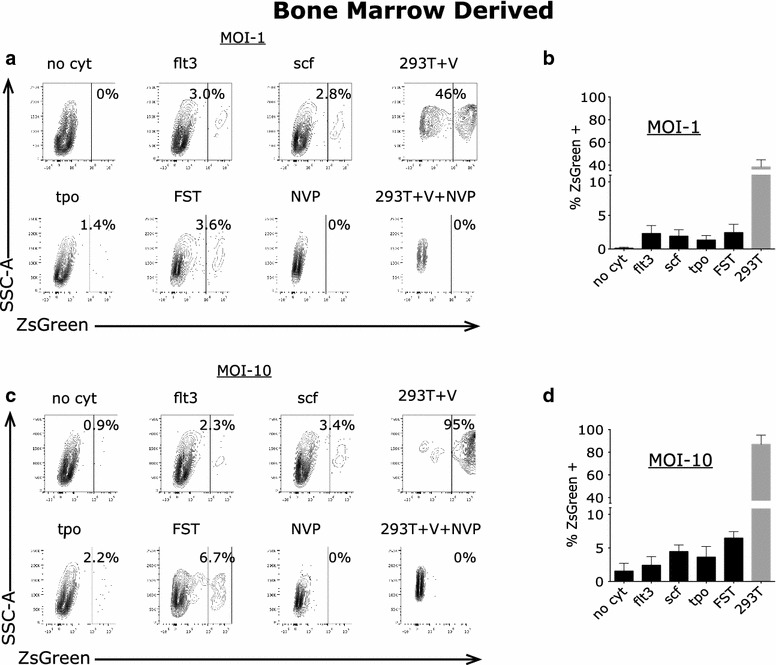
Variable percentages of adult human marrow-derived CD34+ cells express ZsGreen after exposure to pseudotyped HIV-1 vector at relative MOI of 1 in the absence and with incubation with cytokines. Primary human CD34+ cells were incubated with no cytokines (no cyt), Flt3 ligand (FLT3L), stem cell factor (SCF), Thrombopoietin (TPO), all three cytokines (FST) or virus with nevirapine (NVP). 293T cells were similarly incubated with virus (293T + V) or virus plus nevirapine (293 + V + NVP). **a** Cells were evaluated by flow cytometry for expression of ZsGreen after 72 h and a representative example for each condition is shown for viral infection at a MOI of 1. **b** Percent CD34+ cells and 293T expressing ZsGreen with mean values and *error bars* indicating SEM (n = 3) for samples incubated with viral MOI of 1. **c** A representative example for CD34+ cells incubated in each condition is shown for viral MOI of 10. **d** Percent CD34+ cells and 293T cells expressing ZsGreen with mean values and *error bars* indicating SEM (n = 3) for samples incubated with virus at MOI of 10

Less than 1 % of marrow-derived CD34+ cells expressed ZsGreen when incubated in the absence of cytokines and exposed to pseudotyped virus at a MOI of 1 based on titer in permissive 293T cells (Fig. 1). GFP-positive cells only increased to 1–3 % following pre-stimulation with cytokines individually or in combination. In contrast, the permissive 293T cells were efficiently transduced under these conditions, with a large fraction of the population expressing ZsGreen (Fig. 1). A major block to transduction in primary adult human marrow-derived stem cells with or without cytokine stimulation was indicated by these results (p < 0.005 for FST cells compared to 293T cells).

### Transduction of primary adult human marrow-derived CD34+ cells by lentiviral vectors is modestly increased by infection at high MOI

We exposed primary marrow-derived CD34+ cells and 293T cells to pseudotyped virus at a calculated MOI of 10. We observed a modest increase in the transduction efficiency of CD34+ cells under these conditions of high multiplicity with the addition of cytokines. While only 1–2 % of the cells expressed ZsGreen after 72 h without cytokines, 2–16 % of the primary CD34+ cells expressed ZsGreen following the addition of various cytokines (Fig. 1). The vast majority of permissive 293T cells were efficiently transduced under these conditions and expressed ZsGreen. Thus, exposure of the marrow-derived HSPCs to high concentrations of virus resulted in modest increases in the efficiencies of transduction when augmented by cytokine stimulation, particularly in the presence of a cytokine cocktail, but were still significantly decreased compared to 293T cells (p < 0.005 for FST cells compared to 293T cells). These conditions of increased MOI and prestimulation with a cytokine cocktail are similar to those used in many clinical gene therapy protocols [3–5, 7].

### Transduction of primary adult human peripheral mobilized CD34+ cells by lentiviral vectors is inefficient

**Fig. 2:**
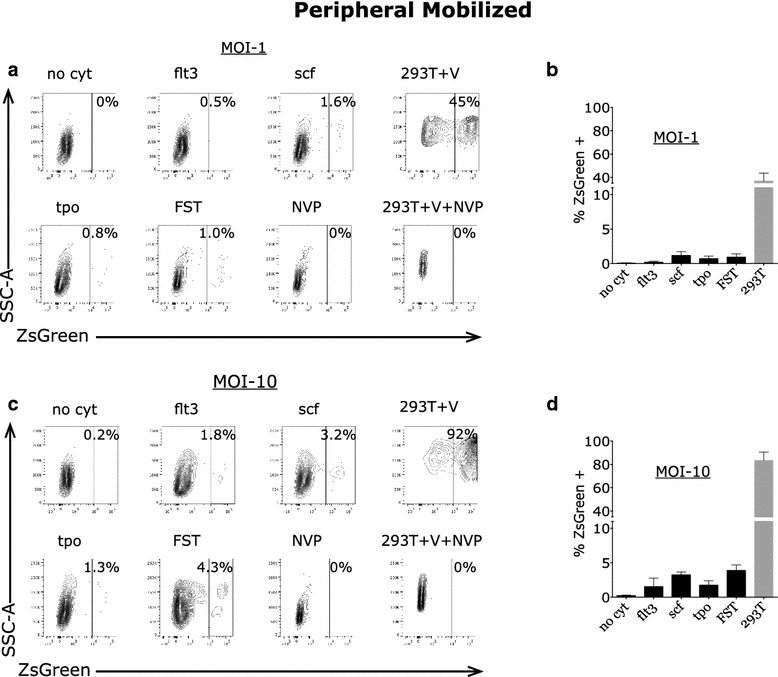
Variable percentages of adult human marrow-derived CD34+ cells express ZsGreen after exposure to pseudotyped HIV-1 vector at relative MOI of 10 in the absence and with incubation with cytokines. Primary human CD34+ cells were incubated with no cytokines (no cyt), Flt3 ligand (FLT3L), stem cell factor (SCF), Thrombopoietin (TPO), all three cytokines (FST) or virus with nevirapine (NVP). 293T cells were similarly incubated with virus (293T + V) or virus plus nevirapine (293 + V + NVP). **a** Cells were evaluated by flow cytometry for expression of ZsGreen after 72 h and a representative example for each condition is shown for viral MOI of 1. **b** Percent CD34+ cells and 293T cells expressing ZsGreen with mean values and *error bars* indicating SEM (n = 3) for samples incubated with viral MOI of 1. **c** A representative example for CD34+ cells incubated in each condition is shown for viral MOI of 10. **d** Percent CD34+ cells and 293T cells expressing ZsGreen with mean values and *error bars* indicating SEM (n = 3) for samples incubated with virus at MOI of 10

Primary adult human peripheral mobilized CD34+ cells, in distinction from bone marrow-derived cells, were next used to study the efficiency of lentiviral transduction under various conditions. When adult human peripheral mobilized CD34+ cells were incubated in the absence or presence of cytokines individually or in combination, and exposed to pseudotyped virus at a MOI of 1, only about 1 % or less of these cells expressed ZsGreen (Fig. 2). In contrast, the permissive 293T cells were efficiently transduced under these conditions (Fig. 2) (p < 0.05 for FST compared to 293T). Thus, as in marrow-derived cells, there is a major block to transduction in primary adult human peripheral mobilized stem cells with or without cytokine stimulation. Under conditions of higher multiplicity (MOI of 10), we observed a modest increase in the transduction efficiency of CD34+ cells to roughly 2–4 % positive cells (Fig. 2). Permissive 293T cells again were efficiently transduced under these conditions (p < 0.005 for FST compared to 293T).

### Intracellular lentiviral DNA forms in primary adult human marrow-derived CD34+ cells exposed to pseudotyped virus at MOI of 1

**Fig. 3:**
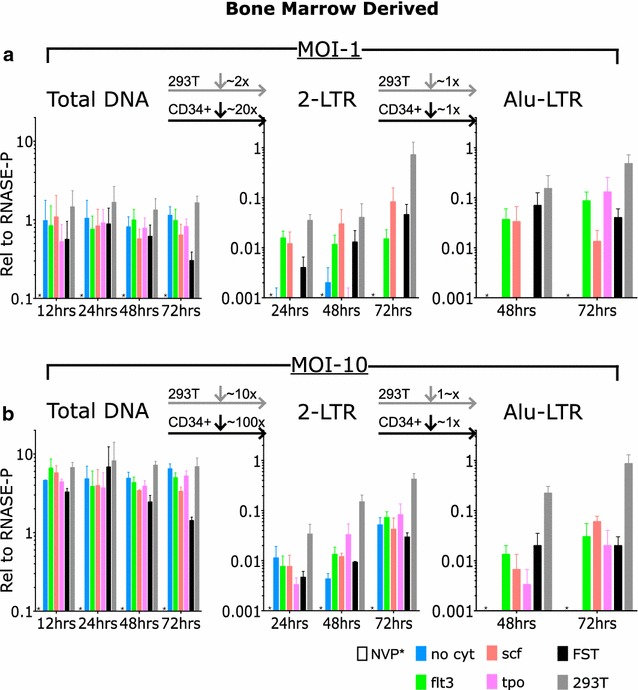
The kinetics of production of total viral DNA, 2-LTR circles, and integrated DNAs in adult human bone marrow derived CD34+ cells after exposure to pseudotyped HIV-1 vector at MOI of 1 and 10. Primary human CD34+ cells were incubated with either nevirapine (NVP) or with no cytokines (no cyt), Flt3 ligand (FLT3L), stem cell factor (SCF), Thrombopoietin (TPO), or all three cytokines (FST). 293T cells were similarly infected as a permissive cell control (293T). **a** Cells were lysed at 12, 24, or 48 h, after infection at MOI of 1, and DNA was extracted and analyzed by taqman PCR analysis for products relative to RNase-P (n = 3 per condition and per time point). DNA levels relative to RNase-P with *bars* representing mean and *error bars* indicating SEM. **b** Cells were analyzed at 12, 24, 48 and 72 h after infection at MOI of 10. Results are shown relative to RNase-P with *bars* representing mean and *error bars* indicating SEM. *Asterisk* indicates undetectable levels with NVP after amplification for 50 cycles

The efficiency of various steps in the course of infection of adult marrow-derived CD34+ cells relative to the efficiency in permissive 293T cells was evaluated using qPCR with established PCR primer sequences, and using the chromosomal RNase-P gene as our Ref. [36]. Levels of total viral DNA, 2-LTR circular DNA, and integrated DNA were evaluated at 12, 24, 48 and 72 h time points (Fig. 3). The DNA copy numbers were calculated relative to the copy number of the RNase-P gene. Infection of the marrow-derived CD34+ cells at an MOI of 1 produced peak levels of total viral DNA in the range of 0.5–1.2 copies relative to the single copy gene, with only minimal change upon incubation with cytokines. Infection of the permissive 293T cells under these conditions yielded a peak mean level of 1.46 copies of total viral DNA per single copy gene (Fig. 3a). Thus, the efficiency of total viral DNA synthesis in CD34+ cells at all time points was almost as high as that seen in permissive cells. Tests of infections carried out in the presence of the RT inhibitor NVP revealed undetectable levels of viral DNA, confirming that the DNA being detected was due to true infection and was not attributable to contaminating plasmid DNA present in the virus preparations. There was apparently no significant block to entry into these cells for virus using the VSV G envelope.

Analysis of circular viral DNAs containing the 2-LTR junction sequence revealed only extremely low levels of DNA in the marrow-derived CD34+ cells (Fig. 3a). Infection at an MOI of 1 produced peak mean levels of 2-LTR circles, relative to the single copy gene, of 0.002 copies with no cytokines, 0.02 with FLT3L, 0.08 copies with SCF, 0.001 copies with TPO, and 0.05 copies with all 3 cytokines combined. The levels in the permissive 293T cells were dramatically higher, at 0.7 copies per single copy standard (p = 0.008 for CD34+ cells relative to 293T cells at 72 h). Across the various conditions, the 2-LTR circular DNAs levels on average were approximately 20 fold lower than the total viral DNA in CD34+ cells, while the circles were only approximately twofold lower than total viral DNA in permissive cells (p = 0.0001 for CD34+ cells peak total DNA versus peak 2-LTR DNA). These resulted were indicative of a strong block in infection of marrow derived CD34+ cells, likely at the stage of nuclear entry.

Analysis of the copy numbers of the integrated proviral DNA in the CD34+ cells revealed a similar reduction as was seen for 2-LTR circles when compared to 293T cells (Fig. 3a). We assessed integration using a taqman-based assay, scoring for linkage between host Alu repeats and the vector LTR sequences (Alu-LTR levels). We could not detect any amplified products in the CD34+ cells after 50 cycles of PCR in the cells not exposed to cytokines. With addition of FLT3L, SCF, TPO, or all three combined (FST), the levels peaked at 0.013–0.13 DNA copies compared to the single-copy standard. The permissive cells yielded approximately 0.48 DNA copies relative to the single-copy gene standard. Thus, although substantial levels of viral DNA were synthesized in the CD34+ cells, very little DNA entered the nucleus to form 2-LTR circles or proviral DNAs.

To confirm our ability to quantify integrated viral DNAs, We performed Alu-LTR PCR on HeLa cell lines transduced at very low MOI and sorted for reporter gene expression, and so carrying single proviruses as controls. We consistently obtained copy numbers of approximately 0.5 relative to RNase-P (in good agreement with expectation).

### Circular and integrated lentiviral DNA forms are also profoundly reduced in primary adult human marrow-derived CD34+ cells exposed to pseudotyped virus at MOI of 10

To explore the simplest approach to increasing transduction of the human CD34+ cells, we repeated the challenges with higher multiplicities of virus. Adult human marrow-derived CD34+ cells and control permissive 293T cells were exposed to pseudotyped virus at a MOI of 10, with and without cytokines, and DNA was extracted at various times post-infection and analyzed by PCR as before. Infection at these high MOIs resulted in substantial increases in the level of total viral DNA over that seen at lower MOI (p = 0.0007 for comparison of peak levels). Infection of the marrow-derived CD34+ cells at an MOI of 10 produced peak mean copies of total viral DNA relative to the single copy gene in the range of 3–7 copies, and with no consistent change upon addition of cytokines (Fig. 3b). In permissive 293T cells peak mean copies of total viral DNA relative to single copy gene were approximately 10 copies. Tests of infections carried out in the presence of the RT inhibitor NVP revealed undetectable levels of viral DNA, again confirming that the DNA being detected was due to true infection and were not attributable to contaminating plasmid DNA present in the virus preparations.

Analysis of circular viral DNAs containing the 2-LTR junction sequence revealed only very low levels of circular DNA in the CD34+ cells (Fig. 3b). Infection of the marrow-derived CD34+ cells at an MOI of 10 produced peak mean levels of 2-LTR circles in the range of 0.03–0.07 copies relative to the single copy gene. Thus across the various conditions, levels of 2-LTR circular DNAs in CD34+ cells were below the levels of total viral DNA by approximately 100 fold, whereas in permissive cells the 2-LTR circle levels were only about tenfold lower than the total viral DNA levels. This indicates a strong block in infection of marrow derived CD34+ cells even at high MOIs, likely at the stage of nuclear entry.

Analysis of the copy numbers of the integrated proviral DNA in the CD34+ cells revealed a similar reduction as seen in 2-LTR circles (Fig. 3b). We could not detect any amplified products in the CD34+ cells after 50 cycles of PCR in the cells not exposed to cytokines. Incubation with cytokines allowed formation of integrated DNA at copy numbers in the range of 0.02–0.06 relative to the single-copy standard. These levels were similar to those seen for 2-LTR circles when cells were exposed to cytokines individually or combined, suggesting that there is no specific block to integration once the DNAs have entered the nucleus in cytokine treated cells; the major block is probably at nuclear entry, and the small amount that is able to enter the nucleus can continue on to integrate.

### Circular and integrated lentiviral DNA forms are reduced in primary adult human peripheral mobilized CD34+ cells

**Fig. 4:**
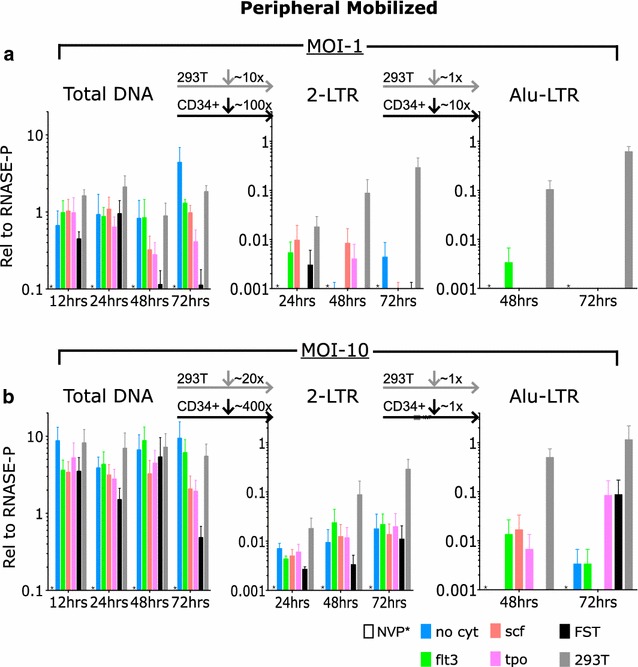
The kinetics of production of total viral DNA, 2-LTR circles, and integrated DNAs in adult human peripheral mobilized CD34+ cells after exposure to pseudotyped HIV-1 vector at MOI of 1 and 10. Primary human CD34+ cells were incubated with either nevirapine (NVP) or with no cytokines (no cyt), Flt3 ligand (FLT3L), stem cell factor (SCF), Thrombopoietin (TPO), or all three cytokines (FST). 293T cells were similarly infected as a permissive cell control (293T). Cells were lysed at 12, 24, or 48, and 72 h after infection at MOI of 1, and DNA was extracted and analyzed by taqman PCR analysis for products relative to RNase-P (n = 3 per condition and per time point). **a** DNA levels relative to RNase-P with *bars* representing mean and *error bars* indicating SEM. **b** Cells were analyzed at 12, 24, 48 and 72 h after infection at MOI of 10. Results are shown relative to RNase-P with *bars* representing mean and *error bars* indicating SEM. *Asterisk* indicates undetectable levels with NVP after amplification for 50 cycles

We performed similar assays for viral DNA levels formed upon infection of primary adult human peripheral mobilized CD34+ cells. In general, the findings were similar to those obtained in the bone marrow-derived cells. Infection of the mobilized CD34+ cells at MOI of 1 produced moderately high levels of total viral DNA (Fig. 4a); only very low levels of circular viral DNA containing the 2-LTR junction (Fig. 4a); and undetectable or extremely low levels of integrated proviral DNA (Fig. 4a). Infections at higher MOI of 10 again produced substantial levels of total viral DNA (Fig. 4b), but only low levels of circular or integrated viral DNAs (Fig. 4b). The data reveal that the incubation of the mobilized cells with the complete cocktail of cytokines led to a dramatic loss of the total viral DNA at the 48 and 72 h time points, especially apparent with infection at low multiplicity (Fig. 4a). The basis for this loss is unclear.

### Increasing amounts of pseudotyped HIV-1 vector improve transduction efficiency but cannot completely overcome the block

**Fig. 5:**
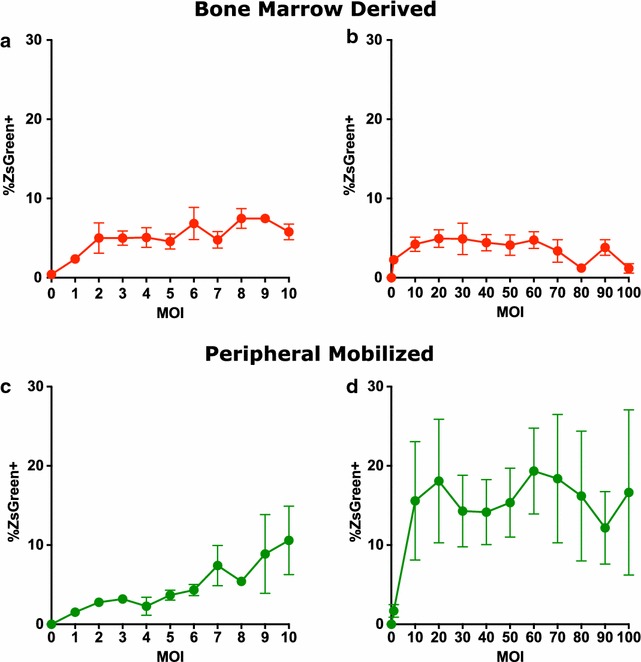
Increasing amounts of pseudotyped HIV-1 vector improve transduction efficiency but cannot completely overcome block. Primary human bone marrow derived and adult peripheral mobilized CD34+ cells were pre-incubated with Flt3 ligand, stem cell factor, and thrombopoietin for 24 h and then exposed to various amounts of virus. **a** Adult bone marrow derived CD34+ cells were exposed to virus at MOI through the range of 0–10 and evaluated by flow cytometry for expression of ZsGreen after 72 h with mean values and *error bars* indicating SEM (n = 3). **b** Adult bone marrow derived CD34+ cells were exposed to virus at MOI through the range of 0–100 and evaluated by flow cytometry for expression of ZsGreen after 72 h with mean values and *error bars* indicating SEM (n = 4). **c** Adult peripheral mobilized CD34+ cells were exposed to virus at MOI through the range of 0–10 and evaluated by flow cytometry for expression of ZsGreen after 72 h with mean values and *error bars* indicating SEM (n = 3). **d** Adult peripheral mobilized CD34+ cells were exposed to virus at MOI through the range of 0–100 and evaluated by flow cytometry for expression of ZsGreen after 72 h with mean values and *error bars* indicating SEM (n = 3)

Primary human bone marrow derived and adult peripheral mobilized CD34+ cells were exposed to increasing amounts of pseudotyped HIV-1 vector and the resulting efficiency of transduction was assessed by flow cytometry evaluation of ZsGreen expression. When adult bone marrow derived CD34+ cells were exposed to increasing viral MOIs between 0 and 10, the numbers of transduced cells increased but quickly leveled off with a maximum level of about 5–10 % (Fig. 5a). Increasing the viral MOI through the range of 0–100 gave some increase in the number of cells expressing ZsGreen, but the values plateaued at about 5 % green at an MOI of about 10 and failed to increase with further increases in virus. There was a drop in number of ZsGreen expressing cells at MOI > 60 (Fig. 5b). When adult peripheral mobilized CD34+ cells were exposed to increasing MOI in the range of 0–10, the number of transduced cells increased steadily up to about 10 % positive (Fig. 5c). At higher MOIs, through the range of 0–100, the fraction of positive cells reached a plateau value of about 20 % at an MOI of 20 and failed to increase with further increases in amount of virus (Fig. 5d).

## Discussion

In murine stem cells (MSCs) the major point of restriction for retroviruses is post-integration and is mediated by proviral silencing [37–39]. In MSCs, following retroviral integration in the host DNA, specific sequence elements of the proviral DNA are recognized by several DNA-binding proteins, resulting in assembly of a silencing complex that permanently silences expression of retroviral genes [31–33, 40–42]. Here we show that in adult human CD34+ HSPCs, transduction is blocked at a different step. Total intracellular viral DNAs were formed efficiently, at levels equal to or even above those seen in 293T cells (Figs. 3, 4). The levels of viral DNA were too low for analysis of the sequences at the viral DNA termini, and thus we cannot rule out the possibility that the linear viral DNAs were not full-length and integration-competent. The major block, however, was seen in the appearance of the nuclear DNA forms. The production of 2-LTR circles and integrated DNA was consistently found to be profoundly reduced in CD34+ cells, to levels approximately 1 % of those seen in permissive 293T cells. Thus, even at high MOI, formation of circles and proviral DNAs was very low in CD34+ cells. The significant reduction in production of 2-LTR circles and integrated DNA suggest that HIV-1 is restricted prior to integration in primary cord CD34+ cells and most likely prior to nuclear entry. The block was consistently observed in multiple independent preparations of CD34+ HSPCs obtained from multiple donors, suggesting that this is a very general property of these cells and not highly variable from individual to individual. The block in the adult cells is similar to that seen in primary cord-derived human CD34+ HSPCs [43], suggesting that restriction prior to integration is a common feature shared by hematopoietic stem cells at all developmental stages.

A close examination of our data would support the idea that there may be multiple points of pre-integration restriction. There is a modest reduction in viral DNA synthesis compared to fully permissive cells. Restriction factors such as Trex1, which degrades RT products, SAMHD1, which depletes NTPs and may degrade viral RNA, and APOBEC3G, which deaminates cytidines and may also block reverse transcription, could all contribute to this restriction. But the more significant point of restriction occurs after viral DNA synthesis, at the time of nuclear entry of the DNA, as evidenced by the low levels of 2-LTR circles relative to permissive 293T cells. This is similar to what we observed in human cord-derived HSPCs. It does not appear that there is restriction at the time of integration per se, as the proviral DNA levels are generally not significantly reduced below that of the 2-LTR circles. The ratio of 2-LTR circles in CD34+ cells to integrated DNA was similar to that of permissive 293T cells, suggesting that there was not a significant intranuclear block as has been demonstrated in quiescent CD4+ cells [44].

Our work also shows that just increasing viral MOI does not fully allow this restriction to be bypassed. Although modest increases in viral exposure enhance the ability to transduce cells it is still only a minority of cells that are successfully transduced even when high viral MOIs are used. Our current work does not allow us to determine whether this plateau is evidence of different permissiveness of subpopulations of CD34+ cells with regard to lentiviral restriction. A MOI of 10–20 resulted in peak transduction and we observed diminished transduction efficiency when MOIs > 60 of our preparations were used. Some components of our preparations, such as the VSV G envelope protein, may be inhibitory or toxic to the cells. The highly purified preparations currently being used in gene therapy trials may achieve higher transduction percentages that are adequate for therapy of some diseases. The use of very high viral MOIs partially overcomes the problem, but better understanding the basic biology may eventually offer alternative solutions.

We did not detect a significant block before reverse transcription, as would be expected if our use of the VSV-G envelope in our pseudotyped virions were significantly limiting cellular entry [45]. The high levels of total DNA also would not support the idea that the restriction we are observing is due to a limitation at the level of cellular entry from an endocytosis defect as has been described in resting CD4+ T cells [46]. Total DNA levels were modestly lower at MOIs of both 1 and 10 for both peripheral mobilized and bone marrow derived CD34+ cells, consistent with prior work, but this was not of the same magnitude as the 20–400 fold decrease that we observed in 2-LTR circles compared to total DNA [47].

We did not see a dramatic improvement in transduction efficiencies, or in formation of circular or integrated DNAs, with preincubation of the cells with any of three cytokines, either individually or all together. There were modest increases in transduction, and the largest increases were often with a cocktail of all three (e.g. Figs. 1d, 2d). The addition of at least one cytokine was necessary for the detection of any integrated DNA at all in marrow-derived (Fig. 3b) or mobilized (Fig. 4b) CD34+ cells, but even with cytokines the levels were still very low. Surprisingly, the addition of the combination cocktail to the mobilized CD34+ cells led to an unusually rapid loss of the total viral DNA at 48 and 72 h time points (Fig. 4a). The basis for this loss is unclear but suggests that transduction in these cells will not tolerate long pre-incubation with cytokines. New culture conditions or other modulators of infection are clearly needed to improve transduction efficiency in these cells.

## Conclusions

HSPCs manifest a strong block to lentiviral infection after viral DNA synthesis and before the appearance of the nuclear DNA forms. While our investigation reveals a strong block prior to appearance of nuclear DNA forms our experiments do not rule out the additional possibility of post-integration silencing in these cells. Understanding the mechanism behind HSPC’s ability to block HIV-1 based viral vectors may potentially be exploited in advancing gene therapy efficiency. It currently remains unclear whether the restriction we are observing in HSPCs is due to a known restriction factor, a novel restriction factor, the absence of a required co-factor, or an innate immune response. The determination that the specific point in the viral lifecycle at which HIV-1 is restricted in human HPSCs is at the time of nuclear entry is an important step toward uncovering the molecular mechanism restricting HIV-1 transduction of HPSCs.

## Methods

### Source of human cells

We obtained primary adult marrow-derived and peripheral mobilized CD34+ cells from AllCells and StemCell Technologies (pre-isolated). All replicates were from different batches of mixed donors.

### Pseudovirus preparation

A modified pnl4.3 HIV-1-based vector env^−^ vpr^−^ nef^−^with ZsGreen replacing luciferase [48] was packaged using 293T cells with a vesicular stomatitis virus glycoprotein (VSV-G) envelope. Culture supernatants were harvested, filtered through a 0.45 μm filter, DNase treated and then concentrated by ultracentrifugation through a 25 % sucrose cushion and resuspended in growth medium. Relative viral MOI titer was determined through infection of the permissive 293T cell line with serial dilutions of the virus preparation.

### Measurement of lentiviral lifecycle stage

DNA isolation was performed using Qiagen DNeasy DNA isolation kits. We used a modified multicolor version of published taqman quantitative polymerase chain reaction (Qpcr) protocols to measure reverse transcription (RT) products, 2-long terminal repeats (2-LTR) and integrated virus (ALU-LTR) replacing mitochondrial genes as a reference with human RNase-P [36]. Primers and probes used were:

- Reverse transcriptase (RT)
- Forward primer: 5′‐TGTGTGCCCGTCTGTTGTGT‐3′
- RT-Reverse primer: 5′‐GAGTCCTGCGTCGAGAGATC‐3′
- RT-Probe: 5-(FAM)‐CAGTGGCGCCCGAACAGGGA-(MGB)-3′
- 2-Long terminal repeat circles (2-LTR)
- Forward primer: 5′‐AACTAGGGAACCCACTGCTTAAG‐3′
- 2-LTR-Reverse primer: 5′‐TCCACAGATCAAGGATATCTTGTC‐3′
- 2-LTR-Probe:5-(FAM)‐TAGTGTGTGCCCGTCTGTTGTGTGAC-(MGB)-3′
- Alu-long terminal repeats (Alu-LTR)
- Forward primer: 5′‐AACTAGGGAACCCACTGCTTAAG‐3′
- Alu-LTR-Reverse primer: 5′‐TGCTGGGATTACAGGCGTGAG‐3′
- Alu-LTR-Probe:5-(FAM)‐TAGTGTGTGCCCGTCTGTTGTGTGAC-(MGB)-3′

*RNase*-*P* copy number reference assay with VIC (5′) TAMRA Quencher (3′) was obtained from Life Technologies. Taqman assays were run for 50 cycles to increase the level at which we report not detected. Where noted in the text and figures that a target was ‘not detected’, product was not amplified during the 50 cycles. ‘Not detected’ implies values less than 1 × 10^−6^ DNA copies per genome based on the cycle at which RNase-P was detected and the possibility that amplification might have occurred after the last cycle at cycle 51.

Expression of fluorophore ZsGreen protein was assessed using flow cytometry on an LSR-II or Fortessa BD Biosciences.

### Cytokine stimulations and stem cell culture

Human HPSCs were cultured in serum free media (X-VIVO 20), and stimulated with different cytokines, SCF, FLT3L, and TPO at 100 ng/ml in each experiment. Pre-incubation was performed for 24 h prior to viral exposure and cytokines were maintained at these levels after viral exposure. Nevirapine was used at 50 μg/ml as a control where noted.

### Viral infection of cells

Target cells were placed in culture and then exposed to specified amount of pseudotyped virus by spinoculation at 37°, 2000 X RPM, for 60 min. Virus was left on the cells and not washed off during subsequent culturing.

### Statistics

Statistical analysis was performed using Prism Software, version 6, for Mac (Graphpad Software Inc., La Jolla, CA, USA). Data are displayed when appropriate as mean plus or minus the standard error of the mean (SEM). Data were compared for statistically relevant differences by using Student’s *t* test with two-tailed analysis.

#### Abbreviations used

HSC, HPC, HSPCs, LTR, HIV-1, MLV, MSCs, IL-3, IL-6, IL-7, SCF, FLT3L, TPO, MOI, siRNA, RT, 2-LTR, Alu-LTR, VSV-G, ZFP809, YY1, Alu, pfu, FACS, TRIM5α, SAMHD1, NTPs, APOBEC3G
